# Methods of Feces Disposal and Fly Prevention in the British Army

**Published:** 1918-10

**Authors:** F. P. Joscelyne


**Disposal of Feces and Fly Prevention in the British Army.** By Major F. P. Joscelyne, A. D. M. S., Sanitation Army Corps.

The speaker said in part :

The disposal of feces has proved to be one of the most difficult problems of sanitation in the war. Different methods have to be taken in forward and back areas. Soil and water level play an important part. Rain, wind, and sunshine increase our difficulties. The fly is at once our deadliest enemy and our best friend in that he immediately informs us of the presence of uncovered feces.

There can be little doubt that dysentery is largely a disease of flies bred on human feces. I will not waste your time to discuss other fly-borne diseases. I will place before you the means we have adopted to dispose of the feces upon which the fly lives and breeds. The B. A. allows 4 o/o of seats.

In the forward area — and by the forward area I include trenches, outposts, and any area within 5000 yards of the enemy line — it is clearly impossible to dispose of feces by incineration. We have left to us only two methods :

1. Sending the feces back to be burned, which has been tried, and has failed.

2. Burying the feces.

The first method of burying feces was by digging a shallow hole in the ground, putting a pole across it, and then throwing a light covering of earth over the deposit. This soon fell out of favor chiefly for the following reasons :

(*a*) The pits were not deep enough to care for more than one, two or three days.

(*b*) Men neglected to cover the feces.

(*c*) Paper flew about all over the place.

(*d*) The latrines were swarming with flies.

(*e*) Even when covered after use, fly maggots were frequently observed burrowing through the earth.

Then uncovered buckets, latrine pails, biscuit tins and oil drums came into use, still with the same pole seat. These were simply emptied into deeper pits and shell holes, were unwashed and subject to the same disadvantages as before.

In 1915 rudimentary seats were, wherever possible, placed over these tins and buckets and matters began to improve. After the advance in the battle of Loos, September 25, 1915, we found the first — as far as I am aware — deep pit latrine. It is probably in existence to this day. The pit was enormous. Speaking from memory I should say that it was 20 feet long, 7 feet wide, and 30 feet deep. It was fitted with an enormous seat, with four or five holes in it, and had no lids to the seats. I say that this was the beginning of deep pit latrines used in trenches, with some reservation. It was the first that I ever saw and I was not long in copying the design. The regiment with which I served built 4 latrines in the front line, 10 feet deep, 2 feet wide, and 6 yards long. We boxed them and lidded them, and they served for about 600 yards of line. They were voted a great success and were used for feces, tins, food refuse, and sometimes for shelter. The back of the box seat was left open for flies to enter and leave as they desired.

The methods now adopted have very considerably improved, and much depends upon the energy and efficiency of the medical officer and upon the amount of discipline driven into troops by the commanding officer. In one line you will find oil drums with close fitting lids. In another line you will find buckets with close fitting lids or boxes, but the method of disposal in both is the same. A careful medical officer will see that his sanitary men keep a liberal supply of cresol in the buckets, and that the feces are buried every night in a shell hole 20 yards behind the trench, and well covered with earth. Too often, owing to carelessness and scanty training of the sanitary staff, cresol is neglected and the feces are ill covered and dumped near the trench. And here our friend, the fly, finds it out, and immediately warns us of its presence.

In another line, where geological conditions are suitable, deep pits are dug and boxed in with fly-proof covers, and, if well looked after, save the sanitary men much trouble and danger. I mention danger because in 1915 I had two of my sanitary men killed while emptying buckets, an operation which necessitated leaving the trench.

In both these systems, now commonly in vogue, I very strongly deprecate the custom of throwing chloride of lime about. My experience is that, when chloride of lime is freely used by a regiment, there is something very wrong with the sanitation of that unit.

Cresol, on the other hand, if properly used in a feces receptacle, is of itself almost sufficient to keep flies away from a latrine. Commanding officers must be made to realize that they are responsible for every branch of sanitation, and that their medical officers are their advisors; at the same time medical officers must be repeatedly reminded to pay more attention to training the men who have to do the manual work. Here, as everywhere, good discipline means good sanitation.

In back areas, where incinerators can be used, the disposal of feces should present fewer difficulties. The ideal method is incineration. It is not always possible, owing to the lack of fuel; and if a plentiful supply of sawdust or wood — coal is out of the question — can not be regularly supplied, then I am all against it. I will tell you what happens : a bucket system is installed at great trouble and expense, and as long as fuel is obtainable everything goes well. Then perhaps the saw mill is moved, and the supply of sawdust is cut off. You struggle on trying to burn, and find yourself landed with an impossible problem to solve. Therefore, I say in forward back areas, if possible, make pit latrines with fly-proof covers, which are adaptable either to pits or buckets, and be ready to use either system at a moment's notice. A well-made pit latrine should be 7 feet deep, and should have a urinal entering into it. It should be covered with well trodden down earth when full to within 3 feet from the surface of the ground. I have found maggots working their way through 2 feet of earth, and this maggot is more dangerous than any other bred fly, since we know that his whole being is a living mass of human feces.

In towns and large villages occupied by troops, there can be no question that incineration is the only permissible method of disposal. Last summer in Poperinghe we were burning 1700 full buckets of mixed feces and sawdust every day. The dump was on the outskirt of the town, with a staff of 14 men. The whole of the refuse of the town, civilian and British, was brought to the dump. The feces was brought in closed iron tanks and dumped into a cement pit; the floor sloped slightly and allowed the urine to drain into large overflow pits, which were emptied twice a week by means of a *vidange* pump, and the liquid put onto the neighboring hop fields. Sawdust was then added to the solid feces, and well mixed by an attendant, who kept his gum boots in a cresol mixture. It was then put in buckets and burned in Horsfalls', and Horsfall type, brick incinerators. Five incinerators were in use, three of them night and day.

In Ypres, during the last winter, I adopted another plan which was equally efficient. Here plenty of wood was available, but sawdust was difficult to get. I built a series of small incinerators, 4 feet by 2 feet, which would just comfortably take a couple of logs on the fire bars. Over the logs was an iron plate, upon which the feces rested and was gradually dried. As much sawdust as was obtainable was mixed with the feces, and the dried mass was used the next morning to light the fire. The whole secret of the plate drying method is to keep your incinerator small. One man can look after 3 of such incinerators; the feces must be added in small quantities, and one incinerator will consume 4 1/2 buckets full of feces in 6 hours.

If you are going to incinerate feces, you must train your men well and not change them about. An untrained man cannot do the work efficiently. For small, out-of-the-way units, I advocate deep pit latrines. For Chinese labor groups and colored troops, and all medical units, I insist on incineration, and use a corrugated iron incinerator, with a drying plate. Inefficient incineration is the worst possible means of dealing with feces. The use of cresol in buckets aids incineration. Every bucket waiting for incineration must be kept covered, and a concrete slab must be built upon which to mix the feces and sawdust. Some of the liquid must be drained off into a urine pit, which should filter from the mixing slab.

Scrupulous cleanliness is necessary in all these operations. And lastly, a dip bowl with soap and towel must be kept handy for the attendant to wash his hands before meals.

Feces burning is not a pleasant occupation and its efficiency would increase if a higher rate of pay were granted for it.

### Fly Prevention.

In the absence of human manure, the fly prefers horse manure to any other substance in which to make his breeding ground. If a manure dump once gets out of hand, it is almost impossible to ever straighten it up again. One finds the utmost difficulty in getting troops to arrange manure in any kind of order.

There are two good methods of stacking manure :

(1) In lines of any length the manure is stacked 10 feet broad, 7 feet high. A trench is dug on either side of it, and the manure is covered with the earth from the trenches to an even depth of perhaps 3 inches of soil. It is just the method that every one knows, who is country bred, of making a vegetable marrow bed. Such a heat arises within that bed that no living thing can ever come out of it. By this method the manure retains its value for some time, and will be of great value to the farms after the war, where cattle are depleted.

(2) The second method of stacking is to fill up old trenches, tread down tight, and cover with earth. By this means an even greater heat is attained, but the disadvantages are :

(*a*) That trenches are not always ready to hand.

(*b*) That much of the manure will be lost to sight.

The next method of disposal is to get farmers to cast it away. It is not very satisfactory because farmers in France and Belgium prefer a rotted manure, and will always leave it, if they can, for weeks on end. Again, they cannot use it on growing crops at the time of year we want most to be rid of it.

Incineration of manure, as far as I have tried it, is a failure, excepting during the sunny weather. A grid is put across a 2 foot trench and the manure laid lightly over it. A fire is started with straw, and the manure will smoulder for days.

Paraffin has been largely used to destroy old dumps. It is extravagant, unsatisfactory, and dangerous. A manure dump is gener -

ally near horse standings, and a night bomber has a good chance of knocking out many horses if he sees a glowing fire.

Two years ago, we were able to get a valuable preparation for spraying farm manures and manure heaps that were impossible to remove. It was called C solution. This solution is not now available, excepting in small quantities.

Manure must be placed as far as possible from dwellings, and clear direction boards must be arranged at every conceivable corner to direct people to the dumps. Every village has its dump and its management is under the direction of the area commandant or town major. Sanitary sections have special instructions to visit these dumps regularly, and a separate heading to report on them in their monthly reports.

These measures have led to a vast improvement during the four years of war, and there are less flies this year than we have seen before.

If manure cannot for any reason be stacked and covered (e. g., in the small farmstead), it is a good plan to cover these fly-breeding haunts with a thin layer of clean white straw. What the action of it is I do not know, but the effect on the diminution of flies is remarkable, and is worth an experiment to those interested. Even one day's trial outside your own billet will convince the most sceptical disbeliever.

Assuming that the fly has now bred in spite of our endeavors to stop him, our next endeavor is to prevent him settling on our food. Wherever possible the windows of messes should have thin muslin, or gauze, or wire gauze tacked over them.

The kitchens should be always — I refer to officers' messes especially — outside the house, and never allowed inside. Officers' kitchens are always the dirtiest seen, and nobody takes the smallest interest in them.

Now comes the great question of food and how to keep it from flies. What happens to our food before we receive it I do not know, but I will give you a brief history of it from the time it is dumped at a forward railway station up to the moment that it is eaten in the front line. Its first resting place after it leaves the truck is mother earth. She may be covered with sail cloth, or not, as the case may be; or possibly the food may be dumped on trench boards. Too frequently, even now, one sees it dumped by the side of a main thoroughfare on the pavement or path. If no cloth or boards are used, the sacking becomes wet, and the glutens or juices ooze into the sacking, the sun comes out, and immediately the flies settle in swarms upon the damp coverings. If it is dusty, every motor lorry showers it with road dust, and again the fly is attracted by the mixture of food and horse manure dust.

Prevention :

(1) Food dumps must have sail cloths or trench boards for the food to rest on.

(2) The food must not be put on the pavements of main roads.

(3) Food should be distributed early in the morning before the fly is awake.

(4) Sanitary officers must visit these dumps at frequent irregular intervals, and take disciplinary action against the officer at fault.

There is no other way that I can see to defeat the fly at this dangerous stage of the food's journey.

The food is now rationed in bulk, and the carts convey it to the Units. Here it receives altogether better treatment because no quartermaster attains his position without merit. I have always found regimental quartermasters most careful in their methods of dealing with food, and a fly in a ration store is rarely seen. This simply means that good discipline lessens the chances of the fly to pollute food.

Here the food is rationed into smaller quantities, and packed in clean sand bags and sacks and sent to the trenches and transport lines. This is done at night and flies have no chance.

In both trenches and transport lines, the food is subjected to great danger of pollution as both of these places generally abound in flies. Here, again, it is simply a matter of discipline whether or not the food becomes contaminated. Scrupulous [cleanliness, as far as possible, must be insisted upon, as inadequate arrangements for the collection of refuse and tins lead almost certainly to dysentery and diarrhea among the troops.

Tanglefoot, wire fly traps, and Japanese wheels are useful in messes and kitchens, but they are of little value if the kitchen is dirty, if the grease traps are not attended to, if the butcher's block is not clean, if the refuse and decaying vegetables are not removed and incinerated, if there is not a covered receptacle for all refuse collected during the day, and if meat safes are not provided and used.

The commanding officer is responsible for all these things being done, and he should be made to realize the responsibilities of disease among the troops entrusted to his care.

